# (5-Fluoro-4′-methyl­biphenyl-3-yl)(2,4,6-tri­methyl­phen­yl)iodo­nium tri­fluoro­methane­sulfonate

**DOI:** 10.1107/S160053681400720X

**Published:** 2014-04-05

**Authors:** Ying Shao, Xiao-Long Liu, Zhu-Hong Wu, Yong-An Xia

**Affiliations:** a Key Laboratory of Fine Petrochemical Engineering, Changzhou University, Changzhou 213164, Jiangsu, People’s Republic of China

## Abstract

In the title mol­ecular salt, C_22_H_21_FI^+^·CF_3_SO_3_^−^, the dihedral angle between the rings of the biphenyl group is 65.6 (1)°. The ring of the mesitylene group is inclined to the fluoro­benzene ring at an angle of 86.1 (3)° and the C—I—C bond angle is 97.0 (2)°. In the crystal, extremely short I⋯O contacts of 2.862 (5) and 2.932 (5) Å occur, due to the strong electrostatic inter­actions between the I atom and two adjacent tri­fluoro­methane­sulfonate counter-ions. There are also C—H⋯F and C—H⋯π inter­actions present: together with the I⋯O bonds, these result in a three-dimensional network.

## Related literature   

For background to di­aryl­iodo­nium salts, see: Grushin (2000 ▶); Merritt & Olofsson (2009 ▶).
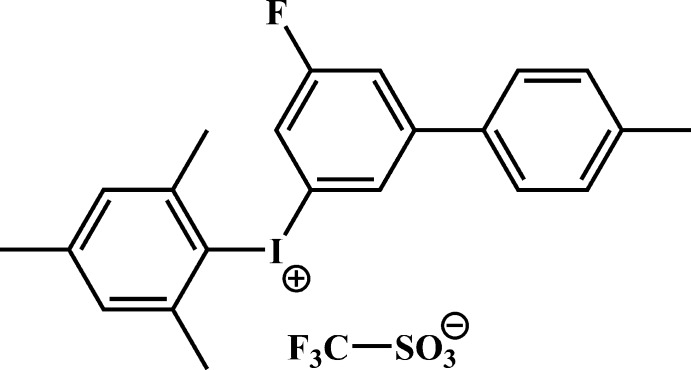


## Experimental   

### 

#### Crystal data  

C_22_H_21_FI^+^·CF_3_O_3_S^−^
*M* *_r_* = 580.37
Monoclinic, 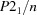

*a* = 9.8987 (8) Å
*b* = 24.374 (2) Å
*c* = 10.0794 (9) Å
β = 105.820 (2)°
*V* = 2339.7 (3) Å^3^
*Z* = 4
Mo *K*α radiation
μ = 1.51 mm^−1^
*T* = 296 K
0.22 × 0.20 × 0.18 mm

#### Data collection  

Bruker APEXII CCD diffractometer
Absorption correction: multi-scan (*SADABS*; Bruker, 2008 ▶) *T*_min_ = 0.732, *T*_max_ = 0.772
13544 measured reflections
4341 independent reflections
3405 reflections with *I* > 2σ(*I*)
*R*_int_ = 0.057

#### Refinement  

*R*[*F* ^2^ > 2σ(*F* ^2^)] = 0.040
*wR*(*F* ^2^) = 0.142
*S* = 1.14
4341 reflections
293 parameters
H-atom parameters constrained
Δρ_max_ = 0.75 e Å^−3^
Δρ_min_ = −1.24 e Å^−3^

### 

Data collection: *APEX2* (Bruker, 2008 ▶); cell refinement: *SAINT* (Bruker, 2008 ▶); data reduction: *SAINT*; program(s) used to solve structure: *SHELXS97* (Sheldrick, 2008 ▶); program(s) used to refine structure: *SHELXL97* (Sheldrick, 2008 ▶); molecular graphics: *SHELXTL* (Sheldrick, 2008 ▶); software used to prepare material for publication: *SHELXTL*.

## Supplementary Material

Crystal structure: contains datablock(s) I, New_Global_Publ_Block. DOI: 10.1107/S160053681400720X/hb7213sup1.cif

Structure factors: contains datablock(s) I. DOI: 10.1107/S160053681400720X/hb7213Isup2.hkl

Supporting information file. DOI: 10.1107/S160053681400720X/hb7213Isup3.cdx

Supporting information file. DOI: 10.1107/S160053681400720X/hb7213Isup4.cml

CCDC reference: 994798

Additional supporting information:  crystallographic information; 3D view; checkCIF report

## Figures and Tables

**Table 1:** Hydrogen-bond geometry (Å, °) *Cg*3 is the centroid of the C14–C19 ring.

*D*—H⋯*A*	*D*—H	H⋯*A*	*D*⋯*A*	*D*—H⋯*A*
C13—H13*A*⋯F1^i^	0.96	2.36	3.193 (9)	145
C22—H22*C*⋯*Cg*3^ii^	0.96	2.76	3.648 (7)	154

Symmetry codes: (i) 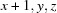; (ii) 
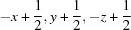
.

**Fig. 1.**
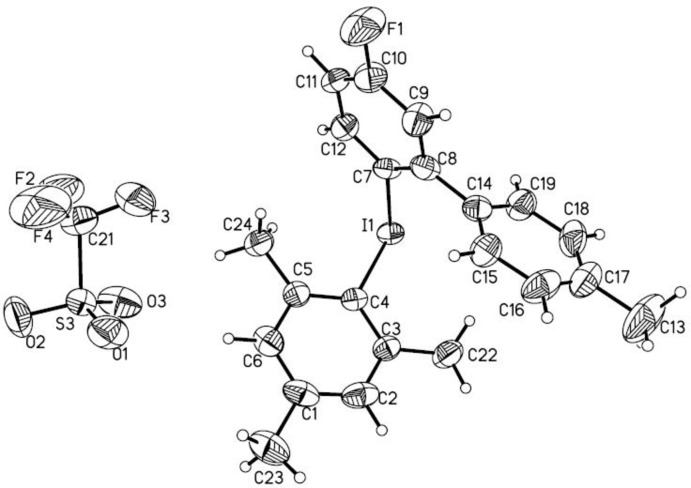
View of the title compound, showing 50% probability ellipsoids.

**Fig. 2.**
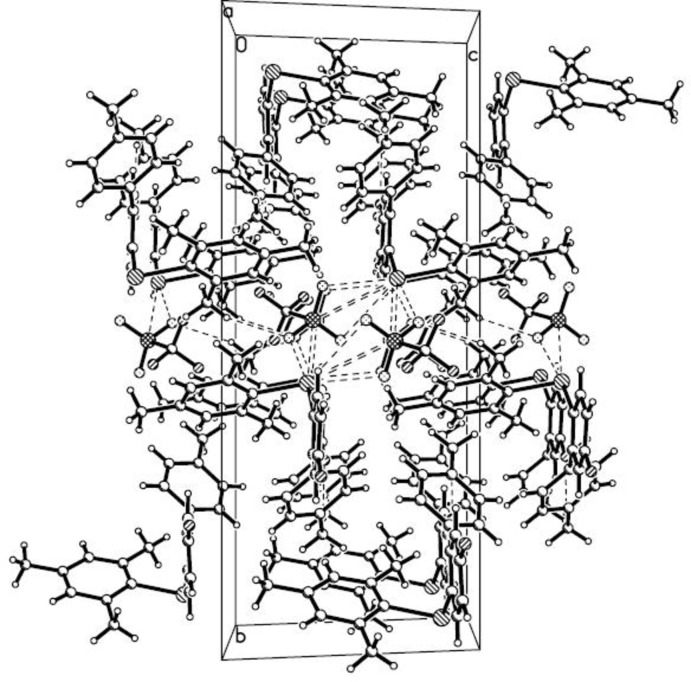
Perspective view of the title compound along a direction. Labels of atoms have been omitted for clarity.

## supplementary crystallographic information

### 1. Comment

The use of diaryliodonium salts has recently gained considerable attention in organic synthesis for arylation of organic bases (Merritt *et al.*, 2009; Grushin *et al.*, 2000). The title compound (Fig. 1) is an important representative of such reagents. In the molecule of the compound, the iodine atom lies almost in the plane of both attached benzene rings with r.m.s. deviations of 0.012 (2) Å and 0.028 (1) Å from the C1—C6 and C7—C12 mean planes respectively. The dihedral angle between the rings of the biphenyl group is 65.6 (1)°. The ring of the mesitylene group is inclined to the phenyl rings of the biphenyl group by 93.9 (2)° (for fluorobenzene ring) and 22.4 (2)° (for toluene ring). Extremely short intermolecular I···O contacts [2.93 (5) and 2.86 (6) Å] occur, due to strong electrostatic interactions between the I atom and two adjacent trifluoromethanesulfonate counter-ions. There are also C—H···F and C—H···π hydrogen bonds present (contact distances are shown in Table 1), which combined with the other inter-actions, form a three-dimensional network (Fig. 2).

### 2. Experimental

*m-*CPBA10 (85%, 2.5 mmol), 3-fluoro-5-iodo-4'-methylbiphenyl (2.0 mmol), and mesitylene (3.0 mmol) were dissolved in CH_2_Cl_2_ (5 ml). Then, TfOH (5.0 mmol) was added to the solution dropwise at 0 °C and the mixture was stirred at room temperature for 2 h and the solution was concentrated *in vacuo*. Et_2_O (1 ml) was added and the mixture was stirred at r.t. for 10 min to precipitate out an yellow solid. The precipitate was filtered off, washed with Et_2_O, and dried under vacuum to give the salt. Yield 76%. Yellow blocks were obtained by slow evaporation of a petroleum / CH_2_Cl_2_ solution.

### 3. Refinement

All the H atoms were placed in geometrically idealized positions and constrained to ride on their parent atoms, with C—H distances of 0.93–0.97 Å, and with *U*_iso_(H) = 1.2*U*_eq_(C).

### Crystal data

**Table:**

C_22_H_21_FI^+^·CF_3_O_3_S^−^	*F*(000) = 1152
*M**_r_* = 580.37	*D*_x_ = 1.650 Mg m^−^^3^
Monoclinic, *P*2_1_/*n*	Mo *K*α radiation, λ = 0.71073 Å
Hall symbol: -P 2yn	Cell parameters from 3546 reflections
*a* = 9.8987 (8) Å	θ = 0–25.5°
*b* = 24.374 (2) Å	µ = 1.51 mm^−^^1^
*c* = 10.0794 (9) Å	*T* = 296 K
β = 105.820 (2)°	Block, yellow
*V* = 2339.7 (3) Å^3^	0.22 × 0.20 × 0.18 mm
*Z* = 4	

### Data collection

**Table:**

Bruker APEXII CCD diffractometer	4341 independent reflections
Radiation source: fine-focus sealed tube	3405 reflections with *I* > 2σ(*I*)
Graphite monochromator	*R*_int_ = 0.057
φ and ω scans	θ_max_ = 25.5°, θ_min_ = 1.7°
Absorption correction: multi-scan (*SADABS*; Bruker, 2008)	*h* = −11→11
*T*_min_ = 0.732, *T*_max_ = 0.772	*k* = −27→29
13544 measured reflections	*l* = −12→12

### Refinement

**Table:**

Refinement on *F*^2^	Primary atom site location: structure-invariant direct methods
Least-squares matrix: full	Secondary atom site location: difference Fourier map
*R*[*F*^2^ > 2σ(*F*^2^)] = 0.040	Hydrogen site location: inferred from neighbouring sites
*wR*(*F*^2^) = 0.142	H-atom parameters constrained
*S* = 1.14	*w* = 1/[σ^2^(*F*_o_^2^) + (0.0805*P*)^2^] where *P* = (*F*_o_^2^ + 2*F*_c_^2^)/3
4341 reflections	(Δ/σ)_max_ < 0.001
293 parameters	Δρ_max_ = 0.75 e Å^−^^3^
0 restraints	Δρ_min_ = −1.24 e Å^−^^3^

### Special details

**Table:**

Geometry. All e.s.d.'s (except the e.s.d. in the dihedral angle between two l.s. planes) are estimated using the full covariance matrix. The cell e.s.d.'s are taken into account individually in the estimation of e.s.d.'s in distances, angles and torsion angles; correlations between e.s.d.'s in cell parameters are only used when they are defined by crystal symmetry. An approximate (isotropic) treatment of cell e.s.d.'s is used for estimating e.s.d.'s involving l.s. planes.
Refinement. Refinement of *F*^2^ against ALL reflections. The weighted *R*-factor *wR* and goodness of fit *S* are based on *F*^2^, conventional *R*-factors *R* are based on *F*, with *F* set to zero for negative *F*^2^. The threshold expression of *F*^2^ > σ(*F*^2^) is used only for calculating *R*-factors(gt) *etc*. and is not relevant to the choice of reflections for refinement. *R*-factors based on *F*^2^ are statistically about twice as large as those based on *F*, and *R*-factors based on ALL data will be even larger.

### Fractional atomic coordinates and isotropic or equivalent isotropic displacement parameters (Å^2^)

**Table:**

	*x*	*y*	*z*	*U*_iso_*/*U*_eq_	
O1	0.3026 (6)	0.06955 (18)	0.8802 (5)	0.0656 (14)	
C1	0.6362 (7)	0.1152 (2)	0.6650 (6)	0.0490 (15)	
C2	0.7058 (6)	0.1336 (2)	0.5718 (7)	0.0533 (16)	
H2	0.7900	0.1524	0.6045	0.064*	
C3	0.6542 (5)	0.1249 (2)	0.4321 (6)	0.0368 (12)	
C4	0.5286 (5)	0.0968 (2)	0.3865 (6)	0.0349 (12)	
C5	0.4560 (6)	0.0757 (2)	0.4748 (6)	0.0375 (12)	
C6	0.5089 (7)	0.0871 (2)	0.6182 (6)	0.0460 (15)	
H6	0.4595	0.0760	0.6799	0.055*	
C7	0.2621 (5)	0.1271 (2)	0.1450 (5)	0.0361 (12)	
C8	0.2661 (6)	0.1846 (2)	0.1484 (6)	0.0419 (13)	
C9	0.1403 (6)	0.2116 (2)	0.1369 (7)	0.0494 (15)	
H9	0.1372	0.2497	0.1361	0.059*	
C10	0.0189 (6)	0.1814 (3)	0.1265 (7)	0.0520 (16)	
C11	0.0135 (6)	0.1253 (2)	0.1185 (6)	0.0456 (14)	
H11	−0.0707	0.1064	0.1055	0.055*	
C12	0.1373 (6)	0.0983 (2)	0.1305 (6)	0.0428 (13)	
H12	0.1382	0.0601	0.1289	0.051*	
C13	0.7364 (8)	0.3215 (3)	0.1208 (12)	0.106 (4)	
H13A	0.8164	0.2994	0.1202	0.159*	
H13B	0.7599	0.3457	0.1990	0.159*	
H13C	0.7092	0.3428	0.0377	0.159*	
C14	0.3908 (6)	0.2173 (2)	0.1483 (6)	0.0387 (12)	
C15	0.4525 (6)	0.2532 (2)	0.2526 (7)	0.0495 (15)	
H15	0.4197	0.2554	0.3305	0.059*	
C16	0.5641 (7)	0.2863 (3)	0.2419 (8)	0.0606 (18)	
H16	0.6043	0.3101	0.3140	0.073*	
C17	0.6170 (6)	0.2852 (3)	0.1295 (8)	0.0584 (18)	
C18	0.5565 (7)	0.2485 (3)	0.0272 (7)	0.0573 (17)	
H18	0.5904	0.2461	−0.0500	0.069*	
C19	0.4453 (7)	0.2149 (3)	0.0362 (7)	0.0523 (15)	
H19	0.4070	0.1905	−0.0349	0.063*	
C21	0.0696 (7)	0.0300 (3)	0.7314 (8)	0.0619 (18)	
C22	0.7369 (6)	0.1458 (3)	0.3363 (7)	0.0537 (16)	
H22A	0.7797	0.1154	0.3026	0.080*	
H22B	0.8086	0.1706	0.3855	0.080*	
H22C	0.6750	0.1647	0.2600	0.080*	
C23	0.6930 (8)	0.1254 (3)	0.8169 (7)	0.071 (2)	
H23A	0.7348	0.0924	0.8617	0.107*	
H23B	0.6179	0.1366	0.8544	0.107*	
H23C	0.7625	0.1539	0.8318	0.107*	
C24	0.3196 (6)	0.0431 (3)	0.4328 (7)	0.0535 (16)	
H24A	0.2414	0.0679	0.4109	0.080*	
H24B	0.3133	0.0197	0.5076	0.080*	
H24C	0.3182	0.0210	0.3535	0.080*	
F1	−0.1015 (4)	0.20869 (18)	0.1145 (6)	0.0883 (15)	
F2	0.0066 (6)	−0.0156 (2)	0.6819 (6)	0.117 (2)	
F3	0.0720 (5)	0.0605 (2)	0.6257 (5)	0.0839 (13)	
F4	−0.0073 (6)	0.0542 (4)	0.7991 (7)	0.147 (3)	
I1	0.44677 (3)	0.080765 (13)	0.17435 (3)	0.03628 (16)	
O2	0.2225 (6)	−0.0155 (2)	0.9503 (5)	0.0728 (14)	
O3	0.3103 (5)	−0.01180 (19)	0.7496 (5)	0.0687 (14)	
S3	0.24551 (15)	0.01636 (6)	0.84096 (15)	0.0418 (3)	

### Atomic displacement parameters (Å^2^)

**Table:**

	*U*^11^	*U*^22^	*U*^33^	*U*^12^	*U*^13^	*U*^23^
O1	0.075 (3)	0.050 (3)	0.060 (3)	−0.018 (2)	−0.002 (3)	−0.007 (2)
C1	0.057 (4)	0.041 (3)	0.039 (3)	0.008 (3)	−0.005 (3)	−0.006 (3)
C2	0.044 (3)	0.042 (3)	0.063 (4)	−0.001 (3)	−0.004 (3)	−0.010 (3)
C3	0.032 (3)	0.035 (3)	0.043 (3)	0.002 (2)	0.009 (2)	−0.003 (2)
C4	0.030 (3)	0.035 (3)	0.037 (3)	0.000 (2)	0.005 (2)	−0.002 (2)
C5	0.031 (3)	0.041 (3)	0.042 (3)	0.009 (2)	0.013 (2)	0.002 (2)
C6	0.046 (3)	0.056 (4)	0.040 (3)	0.015 (3)	0.017 (3)	0.000 (3)
C7	0.030 (3)	0.037 (3)	0.036 (3)	0.003 (2)	−0.001 (2)	0.000 (2)
C8	0.043 (3)	0.034 (3)	0.040 (3)	0.000 (2)	−0.002 (3)	0.001 (2)
C9	0.046 (3)	0.040 (3)	0.068 (4)	0.007 (3)	0.025 (3)	0.003 (3)
C10	0.038 (3)	0.056 (4)	0.067 (4)	0.016 (3)	0.021 (3)	0.009 (3)
C11	0.028 (3)	0.051 (4)	0.054 (4)	−0.003 (3)	0.006 (3)	0.007 (3)
C12	0.041 (3)	0.044 (3)	0.045 (3)	−0.001 (3)	0.013 (3)	−0.003 (3)
C13	0.071 (5)	0.044 (5)	0.203 (12)	−0.015 (4)	0.036 (7)	−0.004 (6)
C14	0.034 (3)	0.038 (3)	0.043 (3)	0.008 (2)	0.008 (2)	0.001 (2)
C15	0.045 (3)	0.052 (4)	0.055 (4)	−0.001 (3)	0.021 (3)	−0.003 (3)
C16	0.046 (4)	0.040 (4)	0.090 (5)	−0.006 (3)	0.009 (4)	−0.014 (3)
C17	0.040 (3)	0.037 (3)	0.100 (6)	0.002 (3)	0.023 (4)	0.005 (3)
C18	0.056 (4)	0.063 (4)	0.066 (4)	0.001 (3)	0.037 (4)	0.011 (3)
C19	0.051 (4)	0.052 (4)	0.054 (4)	−0.008 (3)	0.015 (3)	−0.004 (3)
C21	0.039 (3)	0.074 (5)	0.068 (5)	0.001 (3)	0.006 (3)	0.003 (4)
C22	0.039 (3)	0.061 (4)	0.060 (4)	−0.010 (3)	0.013 (3)	−0.003 (3)
C23	0.083 (5)	0.087 (5)	0.040 (4)	0.007 (4)	0.008 (4)	−0.008 (4)
C24	0.039 (3)	0.056 (4)	0.061 (4)	−0.005 (3)	0.006 (3)	0.011 (3)
F1	0.046 (2)	0.069 (3)	0.155 (5)	0.023 (2)	0.037 (3)	0.017 (3)
F2	0.079 (3)	0.127 (5)	0.115 (4)	−0.059 (3)	−0.024 (3)	0.016 (3)
F3	0.072 (3)	0.094 (3)	0.073 (3)	0.004 (3)	−0.002 (2)	0.029 (3)
F4	0.066 (3)	0.237 (8)	0.137 (6)	0.063 (5)	0.027 (4)	−0.017 (6)
I1	0.0311 (2)	0.0377 (2)	0.0380 (2)	0.00328 (14)	0.00587 (16)	−0.00459 (14)
O2	0.097 (4)	0.078 (3)	0.048 (3)	−0.016 (3)	0.026 (3)	0.014 (2)
O3	0.064 (3)	0.067 (3)	0.066 (3)	0.032 (2)	0.003 (2)	−0.014 (2)
S3	0.0421 (8)	0.0415 (8)	0.0386 (7)	0.0011 (6)	0.0056 (6)	−0.0001 (6)

### Geometric parameters (Å, º)

**Table:**

O1—S3	1.427 (4)	C13—H13B	0.9600
C1—C2	1.383 (9)	C13—H13C	0.9600
C1—C6	1.398 (9)	C14—C15	1.377 (8)
C1—C23	1.501 (8)	C14—C19	1.380 (8)
C2—C3	1.377 (8)	C15—C16	1.395 (9)
C2—H2	0.9300	C15—H15	0.9300
C3—C4	1.384 (7)	C16—C17	1.372 (10)
C3—C22	1.515 (8)	C16—H16	0.9300
C4—C5	1.387 (8)	C17—C18	1.372 (10)
C4—I1	2.107 (5)	C18—C19	1.395 (9)
C5—C6	1.424 (8)	C18—H18	0.9300
C5—C24	1.524 (8)	C19—H19	0.9300
C6—H6	0.9300	C21—F4	1.294 (9)
C7—C12	1.393 (8)	C21—F3	1.304 (8)
C7—C8	1.403 (8)	C21—F2	1.306 (9)
C7—I1	2.099 (5)	C21—S3	1.820 (7)
C8—C9	1.384 (8)	C22—H22A	0.9600
C8—C14	1.469 (8)	C22—H22B	0.9600
C9—C10	1.389 (8)	C22—H22C	0.9600
C9—H9	0.9300	C23—H23A	0.9600
C10—F1	1.342 (7)	C23—H23B	0.9600
C10—C11	1.369 (9)	C23—H23C	0.9600
C11—C12	1.367 (8)	C24—H24A	0.9600
C11—H11	0.9300	C24—H24B	0.9600
C12—H12	0.9300	C24—H24C	0.9600
C13—C17	1.499 (9)	O2—S3	1.416 (5)
C13—H13A	0.9600	O3—S3	1.433 (5)
			
C2—C1—C6	120.0 (5)	C14—C15—H15	119.9
C2—C1—C23	121.4 (6)	C16—C15—H15	119.9
C6—C1—C23	118.6 (6)	C17—C16—C15	122.8 (6)
C3—C2—C1	122.0 (5)	C17—C16—H16	118.6
C3—C2—H2	119.0	C15—C16—H16	118.6
C1—C2—H2	119.0	C16—C17—C18	116.5 (6)
C2—C3—C4	117.7 (5)	C16—C17—C13	121.4 (8)
C2—C3—C22	119.1 (5)	C18—C17—C13	122.1 (8)
C4—C3—C22	123.2 (5)	C17—C18—C19	121.6 (6)
C3—C4—C5	123.2 (5)	C17—C18—H18	119.2
C3—C4—I1	119.4 (4)	C19—C18—H18	119.2
C5—C4—I1	117.4 (4)	C14—C19—C18	121.4 (6)
C4—C5—C6	117.8 (5)	C14—C19—H19	119.3
C4—C5—C24	126.2 (5)	C18—C19—H19	119.3
C6—C5—C24	115.9 (5)	F4—C21—F3	108.4 (7)
C1—C6—C5	119.1 (6)	F4—C21—F2	107.5 (7)
C1—C6—H6	120.4	F3—C21—F2	106.6 (6)
C5—C6—H6	120.4	F4—C21—S3	111.5 (5)
C12—C7—C8	121.6 (5)	F3—C21—S3	111.8 (5)
C12—C7—I1	117.2 (4)	F2—C21—S3	110.7 (5)
C8—C7—I1	121.1 (4)	C3—C22—H22A	109.5
C9—C8—C7	117.0 (5)	C3—C22—H22B	109.5
C9—C8—C14	118.6 (5)	H22A—C22—H22B	109.5
C7—C8—C14	124.0 (5)	C3—C22—H22C	109.5
C8—C9—C10	119.6 (5)	H22A—C22—H22C	109.5
C8—C9—H9	120.2	H22B—C22—H22C	109.5
C10—C9—H9	120.2	C1—C23—H23A	109.5
F1—C10—C11	118.1 (6)	C1—C23—H23B	109.5
F1—C10—C9	118.2 (5)	H23A—C23—H23B	109.5
C11—C10—C9	123.6 (5)	C1—C23—H23C	109.5
C12—C11—C10	117.1 (5)	H23A—C23—H23C	109.5
C12—C11—H11	121.4	H23B—C23—H23C	109.5
C10—C11—H11	121.4	C5—C24—H24A	109.5
C11—C12—C7	121.0 (5)	C5—C24—H24B	109.5
C11—C12—H12	119.5	H24A—C24—H24B	109.5
C7—C12—H12	119.5	C5—C24—H24C	109.5
C17—C13—H13A	109.5	H24A—C24—H24C	109.5
C17—C13—H13B	109.5	H24B—C24—H24C	109.5
H13A—C13—H13B	109.5	C7—I1—C4	97.0 (2)
C17—C13—H13C	109.5	O2—S3—O1	114.7 (3)
H13A—C13—H13C	109.5	O2—S3—O3	115.7 (3)
H13B—C13—H13C	109.5	O1—S3—O3	113.6 (3)
C15—C14—C19	117.4 (5)	O2—S3—C21	104.0 (3)
C15—C14—C8	122.9 (5)	O1—S3—C21	104.1 (3)
C19—C14—C8	119.6 (5)	O3—S3—C21	102.7 (3)
C14—C15—C16	120.3 (6)		
			
C6—C1—C2—C3	−0.4 (9)	I1—C7—C12—C11	176.4 (4)
C23—C1—C2—C3	−179.4 (6)	C9—C8—C14—C15	66.3 (8)
C1—C2—C3—C4	0.0 (9)	C7—C8—C14—C15	−120.6 (7)
C1—C2—C3—C22	−179.4 (6)	C9—C8—C14—C19	−109.7 (7)
C2—C3—C4—C5	−2.2 (8)	C7—C8—C14—C19	63.4 (8)
C22—C3—C4—C5	177.2 (5)	C19—C14—C15—C16	1.1 (9)
C2—C3—C4—I1	−178.6 (4)	C8—C14—C15—C16	−175.0 (5)
C22—C3—C4—I1	0.7 (7)	C14—C15—C16—C17	0.3 (10)
C3—C4—C5—C6	4.6 (8)	C15—C16—C17—C18	−1.4 (10)
I1—C4—C5—C6	−178.9 (4)	C15—C16—C17—C13	179.5 (6)
C3—C4—C5—C24	−178.3 (5)	C16—C17—C18—C19	1.1 (10)
I1—C4—C5—C24	−1.8 (7)	C13—C17—C18—C19	−179.9 (7)
C2—C1—C6—C5	2.9 (8)	C15—C14—C19—C18	−1.4 (9)
C23—C1—C6—C5	−178.2 (6)	C8—C14—C19—C18	174.8 (6)
C4—C5—C6—C1	−4.8 (8)	C17—C18—C19—C14	0.3 (10)
C24—C5—C6—C1	177.8 (5)	C12—C7—I1—C4	−105.0 (4)
C12—C7—C8—C9	0.1 (9)	C8—C7—I1—C4	71.2 (5)
I1—C7—C8—C9	−176.0 (4)	C3—C4—I1—C7	−116.7 (4)
C12—C7—C8—C14	−173.1 (5)	C5—C4—I1—C7	66.6 (4)
I1—C7—C8—C14	10.9 (8)	F4—C21—S3—O2	56.9 (7)
C7—C8—C9—C10	1.7 (9)	F3—C21—S3—O2	178.5 (5)
C14—C8—C9—C10	175.3 (6)	F2—C21—S3—O2	−62.7 (6)
C8—C9—C10—F1	−179.5 (6)	F4—C21—S3—O1	−63.5 (7)
C8—C9—C10—C11	−4.1 (11)	F3—C21—S3—O1	58.1 (6)
F1—C10—C11—C12	179.7 (6)	F2—C21—S3—O1	176.9 (6)
C9—C10—C11—C12	4.3 (10)	F4—C21—S3—O3	177.8 (7)
C10—C11—C12—C7	−2.3 (9)	F3—C21—S3—O3	−60.6 (6)
C8—C7—C12—C11	0.2 (9)	F2—C21—S3—O3	58.2 (6)

### Hydrogen-bond geometry (Å, º)

Cg3 is the centroid of the C14–C19 ring.

**Table:**

*D*—H···*A*	*D*—H	H···*A*	*D*···*A*	*D*—H···*A*
C13—H13*A*···F1^i^	0.96	2.36	3.193 (9)	145
C22—H22*C*···*Cg*3^ii^	0.96	2.76	3.648 (7)	154

Symmetry codes: (i) *x*+1, *y*, *z*; (ii) −*x*+1/2, *y*+1/2, −*z*+1/2.
